# The roles of lipids in SARS-CoV-2 viral replication and the host immune response

**DOI:** 10.1016/j.jlr.2021.100129

**Published:** 2021-09-29

**Authors:** Katherine N. Theken, Soon Yew Tang, Shaon Sengupta, Garret A. FitzGerald

**Affiliations:** 1 Department of Systems Pharmacology and Translational Therapeutics, University of Pennsylvania Perelman School of Medicine, Philadelphia, PA, USA; 2 Institute for Translational Medicine and Therapeutics, University of Pennsylvania Perelman School of Medicine, Philadelphia, PA, USA; 3 Department of Oral Surgery and Pharmacology, University of Pennsylvania School of Dental Medicine, Philadelphia, PA, USA; 4 Department of Pediatrics, University of Pennsylvania Perelman School of Medicine, Philadelphia, PA, USA; 5 Department of Medicine, University of Pennsylvania Perelman School of Medicine, Philadelphia, PA, USA

**Keywords:** lipidomics, lipid metabolism, cholesterol, eicosanoids, phospholipids, sphingolipids, viral infection, COVID-19, coronavirus, SARS-CoV-2, AA, arachidonic acid, ACE2, angiotensin-converting enzyme 2, ARDS, acute respiratory distress syndrome, cysLT, cysteinyl leukotriene, DC, dendritic cell, EPA, eicosapentaenoic acid, IAV, influenza A virus, IPr, IP receptor, LA, linoleic acid, LOX, lipoxygenase, LPL, lysophospholipid, MERS-CoV, Middle East respiratory syndrome related coronavirus, NPC1, NPC intracellular cholesterol transporter 1, PG, prostaglandin, PGI_2_, prostacyclin, RO, replication organelle, RSV, respiratory syncytial virus, S, spike, S1P, sphingosine-1-phosphate, SARS-CoV-2, severe acute respiratory syndrome coronavirus 2, SK, sphingosine kinase, SR-B1, scavenger receptor B type 1, Tx, thromboxane

## Abstract

The significant morbidity and mortality associated with severe acute respiratory syndrome coronavirus 2 infection has underscored the need for novel antiviral strategies. Lipids play essential roles in the viral life cycle. The lipid composition of cell membranes can influence viral entry by mediating fusion or affecting receptor conformation. Upon infection, viruses can reprogram cellular metabolism to remodel lipid membranes and fuel the production of new virions. Furthermore, several classes of lipid mediators, including eicosanoids and sphingolipids, can regulate the host immune response to viral infection. Here, we summarize the existing literature on the mechanisms through which these lipid mediators may regulate viral burden in COVID-19. Furthermore, we define the gaps in knowledge and identify the core areas in which lipids offer therapeutic promise for severe acute respiratory syndrome coronavirus 2.

Severe acute respiratory syndrome coronavirus 2 (SARS-CoV-2) has infected more than 190 million people and accounted for over 4 million deaths worldwide in this pandemic. This public health challenge has underscored the need to limit viral replication and manage the immunological response to infection. Initially thought of as a respiratory pathogen, it is now well accepted that COVID-19 leads to multisystem dysfunction, including considerable cardiovascular, renal, and central pathology, as well as thrombotic and vascular complications.

While the COVID-19 pandemic is a global public health crisis of unprecedented proportions, even before this, seasonal infections with influenza and other viral pathogens were responsible for significant morbidity and mortality. While each pathogen has its unique characteristics that contribute to pathology and host response, they share some common features. Most viral respiratory infections, except for adenoviruses, are caused by RNA viruses. This includes influenza viruses, coronaviruses, respiratory syncytial virus (RSV), human metapneumovirus, parainfluenza viruses, and rhinoviruses. Symptoms might range from mild upper respiratory illness to acute respiratory distress syndrome (ARDS). In most cases, the virus is limited to the upper respiratory tract, spreading to the more distal alveolar epithelium in severe infection. The host must either reduce the pathogen burden (antiviral resistance) or control the adverse effects of the infection on the host's health (disease tolerance) to overcome the infection. Unfortunately, in the process of mounting an effective antiviral response, several proinflammatory signaling cascades are activated. The host may clear the virus only to succumb to the tissue damage that is sustained because of the antiviral response. Host tolerance pathways, on the other hand, may act to suppress the immune response to limit inflammation-induced damage to the host.

Viruses interact with lipid membranes to infect a cell and reprogram lipid metabolism to fuel replication. Furthermore, viral infection stimulates the production of bioactive lipid mediators that mediate the host immune response—both by inducing more inflammation and by regulating the tissue damage. The aim of this review is to summarize our knowledge with a view to elucidating the role of lipids in the pathophysiology of COVID-19, thus highlighting the gaps in knowledge and therapeutic opportunities.

## Lipids in viral entry

**Fig. 1:**
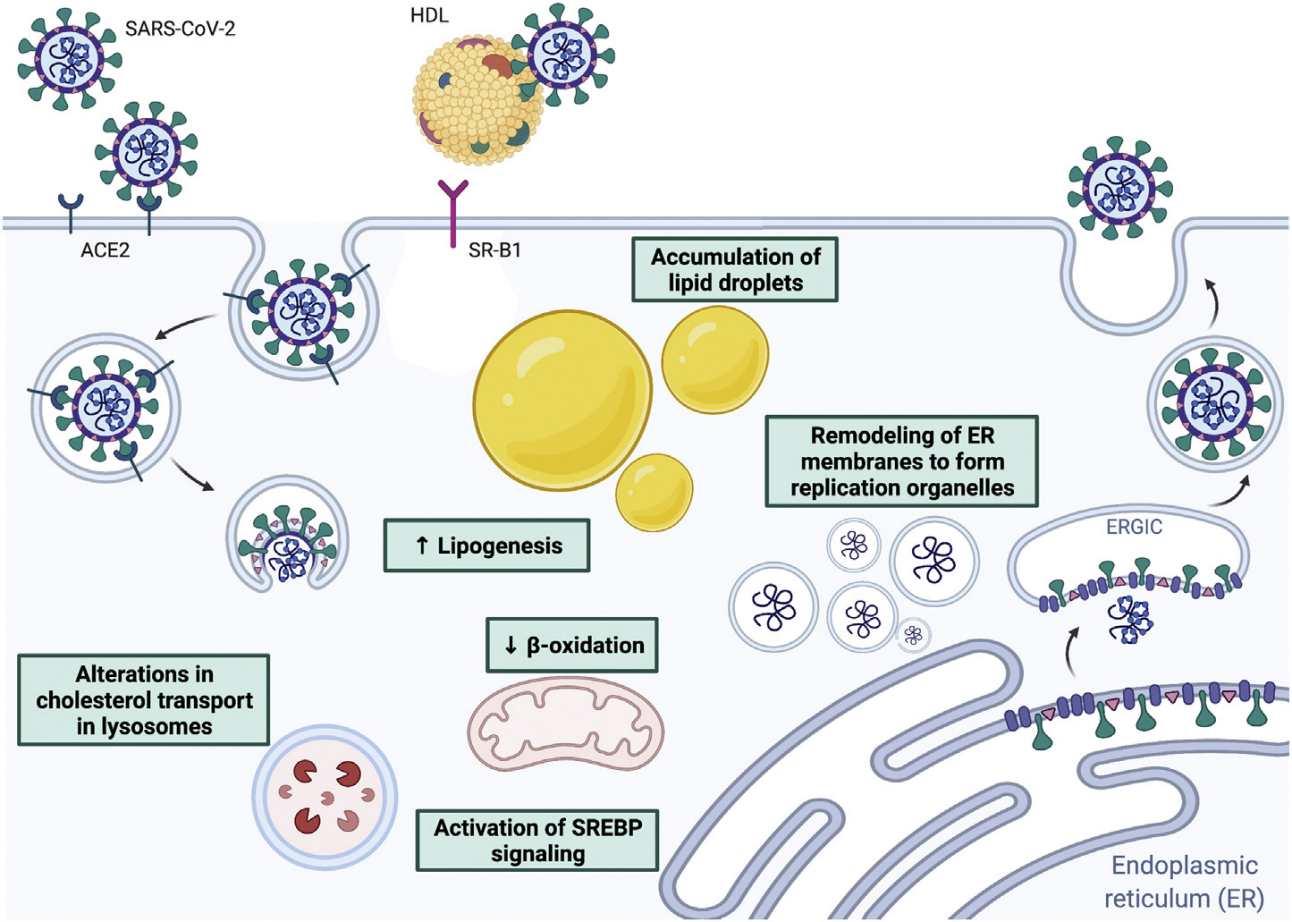
Interactions between coronaviruses and host lipids, including receptor binding and fusion, remodeling of endoplasmic reticulum–derived membranes to form replication organelles, and alterations in lipid metabolism to promote viral replication. Created with BioRender.com.

As obligate intracellular pathogens, viruses interact with host lipids throughout their life cycle (Fig. 1). While many of the pathways involved are specific to a particular virus or family of viruses, others generalize across multiple families of viruses and thus may be potential targets for development of broad-spectrum antiviral agents. There are many reviews summarizing the role of lipids in viral entry and replication (1, 2, 3, 4, 5). Here, we highlight a few examples with emphasis on SARS-CoV-2 and other coronaviruses.

Viruses use various strategies to cross the plasma membrane to enter the host cell. Some nonenveloped viruses can induce membrane lysis, generate pores, or hijack cellular transport vesicles to enter target host cells (6). Other viruses enter host cells via apoptotic mimicry by incorporating phosphatidylserine into their lipid envelopes, as external presentation of this phospholipid is an apoptotic signal for phagocytes to initiate cell clearance (7). Most enveloped viruses infect host cells via either direct fusion of their envelopes with plasma membranes or receptor-mediated endocytosis (8). In the case of SARS-CoV and SARS-CoV-2, viral fusion is initiated by binding of the viral spike (S) protein to angiotensin-converting enzyme 2 (ACE2). This is followed by proteolytic cleavage at the S1/S2 boundary by transmembrane serine protease 2 at the plasma membrane or cathepsins in the lysosome, inducing a conformational shift in the S2 subunit that interacts with the membrane to promote viral fusion (9, 10). For SARS-CoV, it has been shown that the S protein must be palmitoylated to interact with the host membrane and promote viral fusion (11, 12). Similarly, S-acylation of the S protein by ZDHHC20 is essential for SARS-CoV-2 infectivity, stabilizing the S protein and facilitating its interaction with lipid bilayers (13). Thus, the host zDHHC enzymes that catalyze the S-acylation of viral proteins may be potential drug targets (14).

Viruses can also utilize lipoprotein receptors to enter the host cell. This has been extensively studied for hepatitis C virus, which can enter cells via the LDL receptor or the HDL receptor scavenger receptor B type 1 (SR-B1) (1, 15, 16). It has also been reported that the SARS-CoV-2 S protein can bind cholesterol in HDL particles, and that uptake of HDL by SR-B1 facilitates viral entry into cells that coexpress the ACE2 receptor (17). Blocking SR-B1 prevents SARS-CoV-2 infection in multiple cell types in vitro (18).

The success of the fusion process also depends upon the composition of the viral envelope and host membrane because the biophysical properties of the various lipid species affect membrane fluidity and curvature. For example, phosphatidylethanolamine and cholesterol enhance membrane fluidity and promote negative curvature that are critical for viral fusion, while lysophospholipids (LPLs) promote positive curvature and inhibit fusion (19). Thus, agents that modify the lipid content of the viral envelope may have utility as broad-spectrum antivirals. One such compound, LJ001, is a membrane-intercalating photosensitizer that exhibits antiviral activity in vitro. Upon light activation, LJ001 generates singlet oxygen that oxidizes unsaturated phospholipids to enhance membrane rigidity and decrease viral fusion (20). Although both the viral envelope and host cell membranes would become oxidized in the presence of LJ001, the host cell can synthesize new lipids to repair its membrane, while the viral envelope is static, which enhances specificity for the virus and prevents cytotoxicity (21). The utility of LJ001 in vivo is limited because of its need for photoactivation and poor pharmacokinetic properties, but it may serve as a lead compound for the development of novel antivirals with a similar mechanism of action (20). Another potential class of broad-spectrum antiviral compounds that target lipid membranes includes rigid amphipathic fusion inhibitors, nucleoside analogs that intercalate in lipid membranes and inhibit the formation of negative curvature (22). Most importantly, in contrast to fusion inhibitors that target viral proteins (23), fusion inhibitors that act on lipid membranes would theoretically exhibit broader antiviral activity with less potential for development of resistance.

Lipid rafts, discrete membrane microdomains enriched in cholesterol and glycosphingolipids, contain high concentrations of cell surface receptors and can serve as platforms to localize the endocytosis machinery. Consequently, they play an important role in facilitating viral entry (24). Depletion of cholesterol with methyl-β-cyclodextrins decreases viral entry for several coronaviruses, including mouse hepatitis virus (25, 26), avian infectious bronchitis virus (27), and SARS-CoV (28, 29, 30). ACE2 has also been shown to colocalize with lipid rafts in host cell membranes (29, 30), although this has been disputed (28). Although glycosphingolipids can function as coreceptors for some viruses (e.g., HIV (31, 32) and influenza A virus [IAV] (33, 34)), this has not been observed for SARS-CoV-2 or other coronaviruses. Sphingomyelin in lipid rafts can be hydrolyzed by acid and neutral sphingomyelinases to ceramide, a lipid that promotes negative curvature and enhances membrane fluidity. Ceramide molecules self-associate to create ceramide-enriched membrane platforms, which cluster receptors and are involved in cell signaling and membrane trafficking (3, 35, 36). Thus, some viruses exploit ceramide-enriched membrane platforms, as in the case of rhinoviruses that activate acid sphingomyelinase to facilitate entry into the cell (37, 38).

In addition to their role in mediating viral fusion, lipids can affect viral entry by altering conformation of either the host or viral receptor. Inhibition of the serine palmitoyltransferase complex, which catalyzes the first and rate-limiting step in de novo ceramide and sphingolipid biosynthesis, altered the conformation of the murine norovirus receptor (CD300lf) such that the virus was unable to bind and infect the cell. This effect was reversed by addition of exogenous ceramide (39). Similarly, a recent study demonstrated that the SARS-CoV-2 S protein tightly binds the free fatty acid, linoleic acid (LA). This binding stabilizes the S protein in a locked conformation and reduces its interaction with the ACE2 receptor. A similar fatty acid–binding pocket was also observed in SARS-CoV and Middle East respiratory syndrome related coronavirus (MERS-CoV) (40). It has also been reported that omega-3 fatty acids, including LA and eicosapentaenoic acid (EPA), can interact with the receptor-binding domain of the SARS-CoV-2 S protein and inhibit attachment to the ACE2 receptor in vitro (41).

## Lipids in viral replication

Upon entering the host cell, viruses reprogram cellular metabolism to remodel cellular membranes and fuel production of new virions (5, 42). Positive-sense RNA viruses assemble membrane-enclosed replication organelles (ROs), which localize the viral replicase and cofactors in close proximity and may shield the virus from immune recognition (1). The composition and source of lipids to form ROs varies among different families of viruses (2, 5). However, recruitment of phosphatidylinositol 4–kinase IIIβ appears to be a common strategy for remodeling cellular membranes, as this enzyme is important for the formation of ROs for human rhinovirus (HRV, RV-A16) (43), Aichi virus (44), Coxsackie B virus, and hepatitis C virus (45). In contrast, West Nile virus and dengue virus rely on fatty acid synthase to alter the lipid content at the replication membranes (46, 47).

Several studies have demonstrated that coronavirus ROs are derived from the endoplasmic reticulum (48, 49, 50, 51), and for SARS-CoV, the viral nonstructural proteins nsp3, nsp4, and nsp6 are involved in RO formation (52). Recent evidence suggests that cytosolic phospholipase A_2_α, which cleaves phospholipids at the sn-2 position to release LPLs and arachidonic acid (AA), is involved in coronavirus RO formation. Inhibition of cytosolic phospholipase A_2_α decreased LPL release, which decreased the formation of double membrane vesicles and impaired replication of HCoV-299E and MERS-CoV in vitro (53). Similarly, HCoV-299E infection in Huh-7 cells in vitro increased the levels of LPLs, specifically lysophosphatidylcholines and lysophosphatidylethanolamines, and fatty acids, including AA and LA, which the authors attributed to activation of cPLA_2_. Interestingly, addition of exogenous AA or LA decreased replication of both HCoV-299E and MERS-CoV, but it was not determined whether this was due to downstream lipid mediators generated from these fatty acids or activation of the Lands cycle that converts LPLs to phospholipids (54).

Several studies indicate that cholesterol homeostasis plays a critical role in coronavirus infection. MERS-CoV infection in vitro enhanced accumulation of lipid droplets and cholesterol and upregulated genes involved in lipid biosynthesis by activating SREBPs. Inhibition of SREBP DNA-binding activity with AM580 decreased viral replication in vitro. AM580 treatment in vivo decreased viral titers, improved survival, and decreased lung histopathology in mice infected with MERS-CoV or H7N9 IAV (55). Multiple genetic screens have also identified cholesterol biosynthesis as a key pathway in SARS-CoV-2 infection (56, 57, 58, 59, 60). SREBP-2, SREBP cleavage-activating protein, membrane-bound transcription factor site-1 protease, and membrane-bound transcription factor site-2 protease were consistently observed as critical host factors, and other genes linked to cholesterol metabolism and trafficking, including LDL receptor, NPC intracellular cholesterol transporter 1 (NPC1), NPC intracellular cholesterol transporter 2, and ER membrane protein complex subunit 1, were enriched in screens of SARS-CoV-2, HCoV-229E, and HCoV-OC43 infection (56, 59, 60). NPC1 has been shown to play a role in replication for multiple viruses, including Ebola virus, HIV, and Chikungunya virus; thus, NPC1 inhibition has been proposed as a potential antiviral strategy in COVID-19 (61). SARS-CoV-2 infection in monocytes increased lipid droplet formation and upregulated genes involved in lipid metabolism, including CD36, PPAR-γ, SREBP-1, and diacylglycerol acyltransferase-1. Inhibition of diacylglycerol acyltransferase-1 with A922500 dose-dependently reduced viral load in primary human monocytes and inhibited viral replication in Vero E6 cells. Viral particles colocalized with lipid droplets, predominantly associated with the phospholipid monolayer, suggesting that lipid droplets may serve as a replication platform for SARS-CoV-2 (62). Taken together, these results indicate that targeting SREBP signaling might be a viable therapeutic approach to utilize against multiple coronaviruses.

Genetic screens also highlight the importance of lysosomes and autophagy in the SARS-CoV-2 life cycle, potentially via their roles in lipid trafficking and metabolism. In addition to NPC1 and NPC intracellular cholesterol transporter 2, which have well-recognized roles in cholesterol transport in the lysosome, TMEM41B was identified as a critical host factor required for replication of multiple coronaviruses, including SARS-CoV-2 (59, 63). Although this protein has not been well characterized, it has been shown to regulate autophagy and lipid metabolism; TMEM41B-deficient cells exhibit impaired autophagosome formation, enlarged lipid droplets, and reduced β-oxidation of fatty acids (64, 65). In the case of SARS-CoV-2 infection, it has been hypothesized that TMEM41B facilitates ER membrane remodeling to form ROs (59, 63). Sigma-1 and sigma-2 receptors, both of which appear to play roles in cholesterol transport (66, 67), were identified as potential targets in SARS-CoV-2-human protein–protein interaction screens (68, 69). In screens of repurposed drugs, it has been observed that there was a strong correlation between antiviral efficacy against SARS-CoV-2 and the magnitude of phospholipidosis in vitro (70). However, none of the lead compounds tested exhibited significant antiviral activity in vivo, underscoring the need for additional research to translate these in vitro observations into a useful therapeutic approach in humans.

## Lipids in regulating the immune response to viral infection

Eicosanoids are immunomodulatory and may also impact viral replication and the host antiviral response. These lipid mediators include prostaglandins (PGs) and thromboxane (Tx)–together, prostanoids–formed by the PG synthase enzymes, known as cyclooxygenases (COXs)-1 and COX-2; the leukotrienes, hydroperoxy and hydroxy fatty acids formed by the lipoxygenase (LOX) enzymes (5-LOX, 12-LOX, 15-LOX); and epoxyeicosatrienoic acids and 20-hydroxyeicosatetraenoic acid formed by cytochrome P450 enzymes. Targeting these pathways has been proposed as a strategy to dampen cytokine storm and treat complications of SARS-CoV-2 infection (71, 72, 73).

Genetic deletion or pharmacologic inhibition of COX-2 decreases pulmonary inflammation and improves mortality in mouse models of IAV infection (74, 75, 76, 77, 78). Interestingly, mortality was higher in COX-1^−/−^ mice (74) and mice treated with the COX-1 selective inhibitor SC560 (75), relative to controls. However, the mechanism by which selective COX-1 inhibition may worsen outcomes in IAV infection was not investigated, and the relative effects of nonselective versus COX-2 selective nonsteroidal anti-inflammatory drugs have not been delineated. COX-2 inhibition may enhance the early antiviral response after IAV infection in the setting of a chronic inflammatory state such as that induced by obesity (79). Although diet-induced obese mice had higher levels of cytokines and chemokines in lung homogenates at baseline, they displayed poor induction of type 1 interferon responses early in the course of infection and were subsequently unable to clear the virus. Acetaminophen treatment before IAV infection not only restored the early type 1 interferon induction in obese mice but also improved survival on day 14 after infection. Of note, no significant differences in survival due to acetaminophen were observed in lean mice after IAV infection (79).

Of the individual prostanoids, PGE_2_ and PGD_2_ have been most studied in the context of viral infection. Inhibition of PGE_2_ enhanced antiviral immunity and improved survival after IAV infection in mice. Compared with wild-type controls, mice lacking the microsomal PGE synthase-1 (*Ptges*^*−/−*^) had lower viral loads and greater infiltration of macrophages, monocytes, and dendritic cells (DCs) into their lungs and BALF 3 days after infection. After IAV infection in vitro, bone marrow–derived macrophages from *Ptges*^*−/−*^ mice produced more interferon-β and had increased apoptosis than infected wild-type bone marrow–derived macrophages. This difference was abrogated by addition of exogenous PGE_2_, but not other prostanoids, and was mediated through its receptors, EPr2 and EPr4. Furthermore, *Ptges*^*−/−*^ mice exhibited a more robust adaptive immune response, with higher levels of CD4^+^ and CD8^+^ T cells in lymph nodes, BALF, and lung on days 9 and 11 after infection. Finally, pharmacologic inhibition of microsomal PGE synthase-1 or EPr2 and EPr4 signaling in vivo improved survival after IAV infection, and this benefit was lost in mice lacking the interferon-α/β receptor. These results indicate that PGE_2_ suppresses the innate and adaptive immune response to IAV infection in a type I interferon–dependent manner (80). A recent study reported a host-coronavirus protein interaction between PGE_2_ synthase 2 and nsp7 that was conserved among MERS-CoV, SARS-CoV, and SARS-CoV-2 (68), but whether this interaction impacts viral replication has yet to be elucidated.

Several studies suggest that PGD_2_ plays a role in the immune response to respiratory viral infections as well. DPr1 signaling delays migration of DCs to the lung and lymph nodes via downregulation of the chemokine CCR7 (81, 82). Interestingly, the impact of DPr1 signaling and delayed DC migration on adaptive immune responses appears to be age dependent. DPr1 inhibition enhanced DC migration and T-cell proliferation and increased survival in older mice (12 months of age), but not in young mice (6 weeks of age), after SARS-CoV and IAV infection (82). PGD_2_ also contributes to the pathogenesis of RSV bronchiolitis and susceptibility to asthma via DPr2 signaling (83). In a neonatal model of severe RSV bronchiolitis, treatment with a DPr2 inhibitor decreased viral load and improved morbidity via upregulation of IFN-λ. This effect was recapitulated by treatment with a DPr1 agonist, suggesting that these two receptors for PGD_2_ have opposing roles in the regulation of the antiviral response.

Prostacyclin (PGI_2_) can regulate the inflammatory response but has not been well studied in context of viral infection. We examined the role of PGI_2_ in RSV infection using mice lacking the IP receptor (IPr) or overexpressing prostacyclin synthase in alveolar and airway epithelial cells (84). Mice overexpressing prostacyclin synthase in alveolar and airway epithelial cells displayed less weight loss and lower viral titers after RSV infection than the littermate controls. In contrast, mice lacking the IPr lost more weight and had higher viral titers, suggesting that PGI_2_ may enhance the antiviral response and improve viral clearance.

While neither PGF_2α_ nor TxA_2_ have been investigated with respect to modulation of viral clearance or host response to viral infection, they may yet contribute to the consequences of viral infection in the lung. For example, PGF_2α_ accelerates the fibrotic reaction to bleomycin and might contribute to this consequence of viral infection (85). More directly, we have shown that antagonism of the thromboxane receptor prevents evolution of ARDS in a lipopolysaccharide model in sheep (86). In the case of COVID-19, suppression of TxA_2_ formation may have the added benefit of platelet inhibition (87), and multiple trials of low-dose aspirin at vatious stages in patients with COVID-19 are ongoing (Table 1).

**Table 1:** Drugs targeting lipid pathways currently in clinical trials for COVID-19

Drug(s)	Target	Rationale for Use in COVID-19	ClinicalTrials.gov Identifier/Reference
Ibuprofen	COX-1 and COX-2	COX-2 inhibition is anti-inflammatory and improves survival in preclinical models of viral infection. Inhibition of PGE_2_ synthesis enhances the type I interferon response to viral infection.	NCT04334629, NCT04382768
Celecoxib	COX-2	COX-2 inhibition is anti-inflammatory and improves survival in preclinical models of viral infection. Inhibition of PGE_2_ synthesis enhances the type I interferon response to viral infection.	NCT04488081
Low-dose aspirin	Platelet COX-1	Inhibition of TxA_2_ synthesis decreases platelet aggregation, which may prevent thrombotic complications of COVID-19.	NCT04365309, NCT02735707, NCT04703608, NCT04483960, NCT04333407, NCT04324463, NCT04381936, NCT04498273, NCT04368377, NCT04363840, NCT04466670, NCT04808895, NCT04937088, NCT04768179, NCT04410328
Epoprostenol and iloprost	IPr	Prostacyclin analogs promote vasodilation in the pulmonary vasculature, which improve inflammation and oxygenation in COVID-19 patients with ARDS.	NCT04705597, NCT04420741, NCT04445246
BGE-175	DPr1	Inhibition of DPr1 signaling enhances the adaptive immune response to viral infection in preclinical models.	NCT04705597
Montelukast and Zafirlukast	CysLT1R	CysLT1R inhibition is anti-inflammatory and decreases airway hyper-responsiveness after pulmonary viral infection.	NCT04871828, NCT04718285, NCT04695704, NCT04389411, NCT04714515
EPA, DHA, and icosapent ethyl	N/A	Omega-3 fatty acids have anti-inflammatory effects.	NCT04505098, NCT04412018, NCT04460651, NCT04957940, NCT04658433, NCT04495816, NCT04647604, NCT04483271; Arnardottir *et al.* (88)
Statins	HMG-CoA Reductase	Inhibition of cholesterol synthesis may deplete cholesterol in lipid rafts. Statins have pleiotropic anti-inflammatory effects and have been associated with improved outcomes in patients with viral pneumonia.	NCT04472611, NCT04904536, NCT04359095, NCT04801940, NCT04380402, NCT04466241, NCT04952350, NCT04900155, NCT02735707, NCT04333407
Fenofibrate	PPAR-α	PPAR-α agonism may reverse alterations in lipid metabolism induced by SARS-CoV-2 and block viral replication.	NCT04517396, NCT04661930
Evolocumab	PCSK9	PCSK9 loss-of-function genetic variants have been associated with a decrease in inflammatory cytokine response and improved survival in septic shock patients.	NCT04941105
Opaganib	SK2	SK inhibition suppresses viral replication and inhibits the hyperinflammatory response to viral infection.	NCT04467840, NCT04414618
Ozanimod	S1P_1_ and S1P_5_	Activation of S1P signaling restrained cytokine storm, reduced lung pathology, and improved survival in preclinical models of viral infection.	NCT04405102

With regard to the LOX pathway, studies suggest that LTB_4_ is protective, whereas the cysteinyl leukotrienes (cysLTs), LTC_4_, LTD_4_, and LTE_4_, worsen outcomes after viral infection. LTB_4_ elicits antiviral activity in both in vitro and in vivo models of viral infections by promoting release of antimicrobial peptides (89, 90, 91) and stimulating interferon production via activation of the nucleotide-binding oligomerization domain–containing protein 2 pathway (92). In mice infected with IAV, LTB_4_ administration 24 h after infection reduced viral load and lung injury (90). In addition to enhancing the antiviral response, there is also evidence that LTB_4_ promotes disease tolerance to IAV infection. LTB_4_ signaling through the BLTr1 stimulated the release of interferon-α, which decreased the proliferation of inflammatory macrophages and reduced IAV-induced lung pathology (93). In contrast, the LTD_4_ signaling through the CysLTr1 increased susceptibility of type 1 alveolar epithelial cells to IAV infection, and treatment with the CysLTr1 antagonist, zafirlukast, improved survival (94). CysLTr1 blockade decreased airway hyper-responsiveness in mice challenged with RSV (95) and attenuated airway hyper-responsiveness, infiltration of inflammatory cells, and excessive mucus production upon reinfection (96). A retrospective analysis in hospitalized patients with COVID-19 reported that the risk of clinical deterioration, defined as any increase in the COVID-19 ordinal scale value from day 1 to day 3 of hospitalization, was significantly lower in patients treated with montelukast than in patients not receiving montelukast (97). Several prospective clinical trials are underway to explore the utility of CysLTr1 antagonists in treating COVID-19 (Table 1).

The epoxyeicosatrienoic acids possess potent anti-inflammatory properties by attenuating cytokine-induced NF-κB activation and leukocyte adhesion to the vascular wall (98), and inhibition of soluble epoxide hydrolase has been studied as a therapeutic strategy to decrease inflammation in vivo (99). Conversely, 20-hydroxyeicosatetraenoic acid activates NF-κB signaling and induces expression of cellular adhesion molecules and cytokines, thereby promoting inflammation (100). However, the role of these cytochrome P450–derived eicosanoids in regulating the host response to viral infection has not been studied to date (71).

The omega-3 fatty acids EPA and DHA have also been suggested as possible anti-inflammatory therapeutics for COVID-19. The anti-inflammatory effects of omega-3 fatty acids have been attributed by some to the formation of specialized proresolving mediators. However, this is controversial, and we find no evidence of their formation in biologically relevant quantities in humans (101). An observational study reported that a higher omega-3 index, EPA + DHA as a percentage of total erythrocyte fatty acids, was inversely associated with the risk of death in 100 hospitalized patients with COVID-19 (102). Results of a pilot study of EPA + DHA supplementation (103) and a case report of icosapent ethyl treatment (104) in patients with COVID-19 also suggest benefit, and additional prospective clinical trials are ongoing (Table 1).

## Lipidomics of COVID-19

Several studies have performed lipidomic profiling in patients with COVID-19. Although there is heterogeneity among these studies regarding the analytical methods used and the patient populations, some consistent findings have been reported, particularly with regard to serum cholesterol and lipoproteins. Serum triglycerides and VLDL are significantly higher, whereas HDL and LDL are significantly lower in patients with COVID-19 than in age- and sex-matched healthy controls (105, 106, 107, 108, 109). Interestingly, several observational studies have demonstrated that statins may improve outcomes in patients with COVID-19 (110, 111), providing support for future studies to evaluate treatment with these drugs prospectively and investigate the mechanisms underlying such a beneficial effect. Metabolomic and transcriptomic profiling also indicates a shift to fatty acid oxidation in patients with COVID-19 compared with healthy controls, which may indicate a metabolic switch to fuel viral replication (105, 112). However, similar alterations in serum lipoproteins and lipid metabolism have also been reported in patients with trauma (113), ARDS (114), and other infections (115), suggesting that the dysregulation of lipid metabolism observed in patients with COVID-19 reflects a common metabolic shift in response to critical illness, rather than a unique signature of SARS-CoV-2 infection. Most studies report that the levels of HDL and LDL return to baseline after recovery. However, some studies suggest that alterations in lipid metabolism persist even after recovery from COVID-19 (116, 117), and it has been reported that lipid metabolism is dysregulated in survivors of SARS 12 years after infection, compared with age-matched healthy volunteers (118). Thus, longitudinal studies in patients who recover from COVID-19 will be instrumental in determining how long these alterations in lipid metabolism persist or if they are associated with long-term sequelae of infection.

Sphingosine-1-phosphate (S1P), a metabolite of sphingosine that can act intracellularly or via one of five G-protein–coupled receptors (S1P_1_ to S1P_5_), is decreased in patients with COVID-19 compared with healthy controls (119, 120). HDL is the major carrier for S1P in the plasma; thus, this decrease may be a consequence of the decrease in HDL levels in patients with COVID-19, as has been previously reported in patients with sepsis (121). S1P signaling can modulate numerous biological processes, including cell proliferation, apoptosis, and inflammation. In mouse models of IAV infection, activation of the S1P_1_ receptor in the lung endothelium restrained cytokine storm, reduced lung pathology, and improved survival (122, 123, 124, 125), while genetic deletion of S1P_1_ in endothelial cells decreased survival and worsened lung pathology (125).

In addition of the immunomodulatory effects of S1P signaling, inhibition of the sphingosine kinases (SK1 and SK2) that phosphorylate sphingosine to S1P may have antiviral activity. In the context of IAV infection, SK1 and SK2 promote viral replication by modulating NF-κB activation (126, 127, 128). Conversely, overexpression of S1P lyase, which irreversibly cleaves S1P to phosphoethanolamine and hexadecanal, inhibited viral protein synthesis, and decreased IAV viral titers in vitro (126, 129). SK inhibition suppresses viral protein and RNA synthesis by decreasing IKKαβ phosphorylation and NF-κB activation. SK inhibition also decreases activation of the ERK MAPK and PI3K/AKT signaling pathways, leading to decreased phosphorylation of RanBP3 and decreased nuclear export of viral RNP complexes (127). Moreover, treatment with SK2 inhibitor (opaganib) in mice in vivo decreased viral titers, blunted weight loss, and decreased mortality after IAV infection (treated: 50% vs. control: 90%). Similar improvements in survival were also observed with SK1 inhibitor treatment (128). Of note, studies of opaganib in patients with SARS-CoV-2 pneumonia are ongoing (Table 1).

Patients with COVID-19 also exhibited lower levels of glycerophospholipids (107, 119, 120) and higher levels of the corresponding LPLs (107, 120), indicating increased phospholipase A_2_ activation. Moreover, expression of enzymes involved in eicosanoid synthesis (*PLA2G4A*, *PTGS2*, *PTGES3*, *ALOX5*, and *ALOX5AP*) was upregulated in peripheral blood mononuclear cells of patients with COVID-19 (130). Consistent with these observations, higher levels of AA and oleic acid (107) and alterations in eicosanoid profiles (131) have also been reported in patients with COVID-19. A recent report suggests that levels of a secretory PLA_2_ may be a biomarker predictive of severe COVID-19 (132). Further investigation is warranted to determine whether drugs that modulate eicosanoid lipid mediators might have antiviral activity in COVID-19.

## Conclusions

The integral role of lipids in the viral life cycle suggests that targeting these pathways may be a viable therapeutic strategy. However, development of such novel antiviral agents for COVID-19 will require a better understanding of the effects of SARS-CoV-2 infection on lipid metabolism in vitro and in model organisms. Furthermore, serial lipidomic analyses in individuals with COVID-19 may identify specific lipid pathways that mediate the heterogenous response to viral infection, serve as prognostic biomarkers, or contribute to long-term sequelae.

## Conflict of interest

The authors declare that they have no conflicts of interest with the contents of this article.
